# Spontaneous thoracic ventral spinal subdural hematoma mimicking a tumoral lesion: a case report

**DOI:** 10.1186/s13256-015-0562-3

**Published:** 2015-06-06

**Authors:** Yong-Jian Zhu, De-Qing Peng, Fang Shen, Lin-Lin Wang, Zhu-Xiao Tang, Jian-Min Zhang

**Affiliations:** 1 Department of Neurosurgery, Second Affiliated Hospital of Zhejiang University School of Medicine, Hangzhou, 310009 China; 2 Department of Neurosurgery, Ningbo No.2 Hospital, Ningbo, Zhejiang 315010 China; 3 Department of Physiology, Zhejiang University School of Medicine, Hangzhou, 310009 China

**Keywords:** Acute paraplegia, Laminectomy, Spinal subdural hematoma

## Abstract

**Introduction:**

Spinal subdural hematoma is rare and can cause serious neurological symptoms. Sometimes, idiopathic spinal subdural hematoma can spontaneously occur without any identifiable underlying etiologies. In this report, we present such an uncommon case of paraplegia caused by idiopathic spinal subdural hematoma that was successfully managed by laminectomy.

**Case presentation:**

A 45-year-old Chinese woman presented with sudden onset of progressive asthenia and numbness in both lower extremities, accompanied by difficulty in micturition. An initial non-contrast spinal magnetic resonance imaging at a local hospital suggested a spinal subdural tumoral hematoma at the T9 level. She was referred to our hospital and an emergency laminectomy from T8 to T10 was performed 22 hours after onset of her initial symptoms. However, nothing but a hematoma was identified during the operation, and a final diagnosis of spontaneous acute spinal subdural hematoma was concluded. She had partial return of sensations and voluntary movement after the operation.

**Conclusions:**

On imaging findings, spinal subdural hematoma could manifest as focal and independent from the dura matter, and, therefore, it should be included in the differential diagnosis of medullary compressive lesions.

## Introduction

Spinal hemorrhages include epidural, subdural and intramedullary hemorrhage, of which spinal subdural hematoma (SSDH) is the least common form. SSDH usually manifests as a sudden onset of back pain and lower limbs paralysis. On a magnetic resonance imaging (MRI) scan of the spine, SSDH is usually hyperintensive on T2-weighted images and isointensive on T1-weighted images in acute phase [1,2]. Its shape could either be disseminated or localized as a round lesion that compresses the afflicted spinal cord, making differential diagnosis between hematoma and tumor difficult. The etiologies of SSDH include vascular lesions, tumors, or anticoagulant treatments, and sometimes it can also result from invasive procedures such as lumbar puncture, epidural anesthesia or even by acupuncture [3-5]. The occurrence of spontaneous SSDH without the aforementioned underlying conditions is even rarer and only 20 cases have been reported in previous literatures [6]. In this report, we present such an uncommon case of paraplegia caused by idiopathic SSDH.

## Case presentation

**Figure 1:**
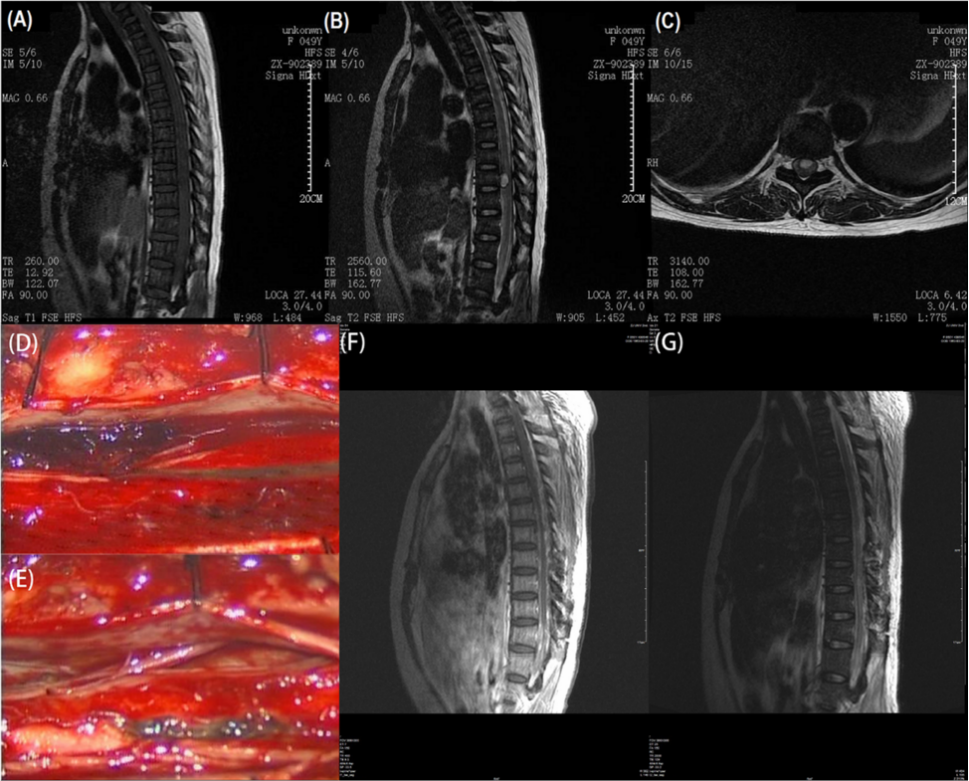
Radiological and intraoperative findings of the presented case of spontaneous acute spinal subdural hematoma. T1-weighted magnetic resonance imaging showed an isointensive lesion situated at the T9 level, ventral to the spinal cord **(A)**. T2-weighted magnetic resonance images showed the lesion was a well-demarcated, oval-shaped extramedullary mass at the T9 level **(B, C)**. An emergency laminectomy from T8 to T10 was performed and only a hematoma was found during the operation **(D)** and it was successfully evacuated **(E)**. Postoperative sagittal T1-weighted contrast enhanced **(F)** and T2-weighted **(G)** magnetic resonance imaging showed total removal of that hematoma.

A 45-year-old Chinese woman presented with sudden onset of progressive asthenia and numbness in both lower extremities, accompanied by difficulty in micturition; she was first sent to a local hospital. Five hours later, she was unable to walk. She reported no fever, back pain, or trauma history. She had no past medical history of note and was on no medication. A general physical examination performed on admission revealed increased heart rate (94 beats per minute) and blood pressure (193/104mmHg). A neurological examination revealed a normal mental state, but grade 0 myodynamia and paresthesia in both lower limbs. An initial non-contrast MRI scan of her spine performed at the local hospital showed a well-demarcated, oval-shaped extramedullary lesion located at the T9 level, ventral to her spinal cord. The lesion appeared isointensive on T1-weighted images and hyperintensive on T2-weighted images, but gadolinium was not administrated in this scan (Figure 1A-C). She was then diagnosed with acute tumoral SSDH and received conservative treatments. However, because of no sign of alleviation, she was referred to our hospital 20 hours after the onset of initial symptoms.

On admission to our department, a neurological examination revealed that her muscle strength was grade 0 in both lower limbs, and grade 5 in both upper limbs. There was a significant loss of coordination in all fine and gross motor movements in both lower limbs, together with a complete absence of proprioception. Sensations like fine touch, pain, temperature and vibration were completely diminished below the umbilical level. Deep tendon reflexes, for example knee and ankle jerk, were negative, while biceps and triceps reflexes were normal. Bilateral Babinski’s signs were positive and muscle tone was normal. In view of progressive deterioration in her neurologic conditions, we performed an emergency laminectomy from T8 to T10 to decompress her spinal cord 22 hours after onset of the initial symptoms. Only a hematoma was found during the operation (Figure 1D & E) and postoperative MRI confirmed the complete removal of the hematoma (Figure 1F & G). Several days after the operation, her tactile anesthesia and proprioception improved to the level that she could correctly identify which toe was moved by others. She had partial return of voluntary movement in her ankles 2 weeks later. Postoperative angiography showed no vascular lesions. She then went to a local hospital for rehabilitation therapy.

## Discussion

Extramedullary subdural hemorrhages or SSDHs tend to happen in the thoracic segment and usually present with sudden back pain that radiates to the upper or lower extremities or to the trunk [7]. Within hours, complete sensory and motor deficits of the medulla or cauda equina follow. These neurological deficits can be severe and progressive. The duration from the onset of back pain to the development of paraplegia is 10 to 26 hours [7]. In general, MRI is helpful in terms of differentiating between tumors and vascular malformations as the underlying etiology [8]. Usually these subdural lesions are convex and crescentic in appearance and tend to be around the spinal cord with delineation of the dural sac and intact epidural fat. Up and down extension is frequent, sometimes extension to the posterior cranial fossa may even be seen. However, the unusual imaging appearance of this presented subdural lesion (focal and independent from the dura) misled the initial radiological diagnosis to be subdural tumoral hematoma. However, in view of the absence of identifiable underlying causes by both intraoperative and postoperative angiographic findings, a diagnosis of idiopathic SSDH was finally reached.

Since accurate diagnosis and prompt treatment strategy (that is, conservative or surgical intervention) are important to a patients’ prognosis, special attention should be paid to those patients with SSDH who present untypical symptoms in future clinical practice. Although there are still some debates on the surgical indications for SSDH, we think emergency surgical hematoma evacuation at the local hospital or more prompt referral might be more preferable than simply conservative treatments. In fact, there is no consensus on the optimal management strategy for those patients with SSDH; strategies range from conservative treatments like intravenous methylprednisolone (pulse) therapy [9], to percutaneous drainage and surgical decompression. We chose laminectomy because our patient experienced progressive neurological deterioration. Moreover, patients’ preoperative neurologic deficits seem to be critical for their prognosis [10,11]. In the present case, surgical removal of the hematoma was performed after 22 hours, which might account for the poor recovery of her muscle strengths in her lower limbs. Although cases that were successfully managed by conservative treatment have been reported [7,12,13], the majority of the patients in the literature had surgical decompression instead [6].

## Conclusions

Although preoperative images of the presented case suggest it to be an extramedullary intradural lesion, for example arachnoid cyst or tumoral lesion with proteinaceous or bloody fluid in it, the intraoperative surgical findings confirmed the diagnosis of idiopathic SSDH. Therefore, SSDH should be included in the differential diagnosis of those lesions that compress the spinal cord.

## Consent

Written informed consent was obtained from the patient for publication of this case report and accompanying images. A copy of the written consent is available for review by the Editor-in-Chief of this journal.
